# Generation and Characterization of a Mouse-Adapted Makona Variant of Ebola Virus

**DOI:** 10.3390/v11110987

**Published:** 2019-10-26

**Authors:** Mable Chan, Anders Leung, Bryan D. Griffin, Robert Vendramelli, Nikesh Tailor, Kevin Tierney, Jonathan Audet, Darwyn Kobasa

**Affiliations:** 1 Special Pathogens Program, National Microbiology Laboratory, Public Health Agency of Canada, 1015 Arlington Street, Winnipeg, MB, R3E 3R2, Canada; mable.hagan@canada.ca (M.C.); anders.leung@canada.ca (A.L.); bryan.griffin@canada.ca (B.D.G.); robert.vendramelli@canada.ca (R.V.); nikesh.tailor@canada.ca (N.T.); kevin.tierney@canada.ca (K.T.); jonathan.audet@canada.ca (J.A.); 2 Department of Medical Microbiology and Infectious Diseases, University of Manitoba, 745 Bannatyne Avenue, Winnipeg, MB, R3E 0J9, Canada

**Keywords:** Ebola virus, Makona, Mayinga, mouse-adapted, animal model, mice, reverse genetics, pathogenesis, VP24

## Abstract

Ebola virus (EBOV) is a zoonotic pathogen that poses a significant threat to public health, causing sporadic yet devastating outbreaks that have the potential to spread worldwide, as demonstrated during the 2013–2016 West African outbreak. Mouse models of infection are important tools for the development of therapeutics and vaccines. Exposure of immunocompetent mice to clinical isolates of EBOV is nonlethal; consequently, EBOV requires prior adaptation in mice to cause lethal disease. Until now, the only immunocompetent EBOV mouse model was based on the Mayinga variant, which was isolated in 1976. Here, we generated a novel mouse-adapted (MA)-EBOV based on the 2014 Makona isolate by inserting EBOV/Mayinga-MA mutations into the EBOV/Makona genome, followed by serial passaging of the rescued virus in suckling mice. The resulting EBOV/Makona-MA causes lethal disease in adult immunocompetent mice within 6 to 9 days and has a lethal dose (LD50) of 0.004 plaque forming units (PFU). Two additional mutations emerged after mouse-adaptation in the viral nucleoprotein (NP) and membrane-associated protein VP24. Using reverse genetics, we found the VP24 mutation to be critical for EBOV/Makona-MA virulence. EBOV/Makona-MA infected mice that presented with viremia, high viral burden in organs, increased release of pro-inflammatory cytokines/chemokines, and lymphopenia. Our mouse model will help advance pre-clinical development of countermeasures against contemporary EBOV variants.

## 1. Introduction

Since its first discovery in 1976, Ebola virus (EBOV) has been responsible for 18 outbreaks with case fatality rates (CFR) ranging from 25–88% [1]. From 2013 to 2016, the largest Ebola virus epidemic in history occurred in West Africa resulting in over 28,000 confirmed cases and more than 11,000 deaths [2]. This outbreak originated in Guinea and within half a year, the virus quickly spread to the capitals of neighbouring countries (Liberia and Sierra Leone) and later, worldwide as a consequence of imported travel-related cases [3]. The causative agent of this outbreak was a new EBOV variant called Makona [4]. 

Despite the resolution of the West African outbreak, the risk of future EBOV outbreaks continues to be a concern. This is exemplified by the ongoing outbreak that is occurring in the Democratic Republic of the Congo (DRC), where 3210 confirmed cases of EBOV and 2144 deaths (~67% CFR) have been reported as of October 10, 2019 [5]. Ebola virus disease (EVD) causes severe, often fatal hemorrhagic fever in humans and is transmitted human-to-human through direct contact with blood and other bodily fluids [1]. Furthermore, EBOV is known to persist in immune-privileged sites of survivors, semen, and breast milk [6,7,8]. Sexual transmission of EVD has been documented to occur months to years post-recovery [9,10]. The possibilities of relapse in survivors of EVD highlights the challenges of monitoring cases of persistent infection and the control of the disease.

There is currently no licensed treatment for EVD; however, a number of experimental vaccines and drugs that are highly effective in animal models are being tested in clinical trials. The monoclonal cocktail (ZMapp), which provided complete protection in non-human primates (NHPs) [11,12], was administered to EBOV patients during the West African outbreak [13] and has been tested in clinical trials [14]. Ebola virus vaccines that are currently being tested in clinical trials include the recombinant vesicular stomatitis virus-based vaccine (rVSV-ZEBOV) and the chimpanzee adenovirus type 3-based (chAd3-EBO-Z) vaccine [15]. Two new drugs, REGN-EB3 and mAB114, have recently shown efficacy at providing protection to EBOV patients from the current Ebola virus epidemic in the DRC [16,17]. 

Animal models are important tools to study viral pathogenesis, transmission, and for the development of therapeutics and vaccines. While non-human primates (NHPs) recapitulate human EVD with high fidelity, they are highly sentient and are best suited as a final animal model for evaluating promising interventions before human trials, after preliminary efficacy has been demonstrated in small animal models. In addition, there are benefits of using NHPs to study viral pathogenesis—for example, studies that examine mechanisms used by the virus to propagate and cause disease, which may lead to the discovery of new targets for antiviral development. Mice are well-suited and the model is a well-defined model for initial research purposes as their genetics are highly characterized and reagents to study mouse immune responses are readily accessible. Wild-type EBOV does not cause disease in immunocompetent mice [18]. Alternatively, immunocompromised mice are susceptible to EBOV infection [19,20,21]; however, the immune deficiencies of these mice limit their use for vaccine and therapeutic studies that rely on the development of host immune responses to provide protection against EVD. To circumvent this problem, EBOV/Mayinga was adapted in mice to cause lethal infection through nine serial passages in progressively older suckling mice [22]. The EBOV/Mayinga-mouse-adapted (MA) virus was shown to be lethal in adult immunocompetent BALB/c, C57BL/6, and CD-1 mice [20]. Disease progression of EBOV/Mayinga-MA resembles that of EBOV/Mayinga in NHPs, where high levels of viral replication are present in blood and multiple tissues (primarily the liver and spleen), and other associated clinical signs of disease such as lymphopenia, thrombocytopenia, and liver and kidney dysfunction are observed [23]. The mouse model does have some shortcomings, including a lack of coagulopathy or hemorrhagic manifestations, or the typical petechial rash that occurs in humans and NHPs [24,25,26]. However, coagulopathy in mice appears to differ depending on the strain of mouse used [27].

Mouse-adapted versions of other filoviruses have been generated by passaging in immunodeficient mice or in combination with immunocompetent mice, including the two strains of Marburg virus (MARV)—Angola [28] and Ravn [29]. However, for EBOV, only the Mayinga variant from 1976 has been mouse-adapted. To provide a useful tool to study countermeasures and pathogenesis of more recent EBOV isolates, we generated a mouse-adapted version of EBOV variant Makona C07, a 2014 isolate from the West African outbreak. In this study, we first inserted mutations that were associated with mouse-adaptation from EBOV/Mayinga-MA into the EBOV/Makona genome to produce an EBOV/Makona-preMA genome. After eight passages of EBOV/Makona-preMA in suckling mice, we produced an EBOV/Makona-MA that was capable of causing lethal disease in adult immunocompetent BALB/c mice. Sequencing of this virus revealed two additional mutations in the nucleoprotein gene (NP, nucleotide position 2357; amino acid H630N) and the membrane-associated viral protein VP24 (nucleotide position 10,979; amino acid K212M) that were acquired during passaging. Using reverse genetics, the role of each mutation on the virulence of EBOV/Makona-MA was investigated. We found that only the 10,979 mutation is critical for EBOV/Makona-MA virulence. Furthermore, rapid viremia, high replication in target organs such as the liver and spleen, lymphopenia, and increased levels of pro-inflammatory cytokines were observed in both EBOV/Makona-MA and EBOV/Mayinga-MA infected mice. The newly generated EBOV/Makona-MA will serve as a useful tool to help advance the field of therapeutics, studies on viral pathogenesis, and persistence in immunoprivileged sites. 

## 2. Materials and Methods 

### 2.1. Viruses

The EBOV Makona C07 virus originated from a serum sample from a human patient from the Ebola virus outbreak in Guinea in 2014 [4]. The virus was cultured as a passage 1 (P1) stock in Vero E6 cells from the clinical specimen. The complete genome of the P1 stock was sequenced, accession KJ660347.2, and this sequence was used to generate the reverse genetics system for EBOV Makona C07 (described in [21]). For all the animal experiments, we used the P2 stocks of our recombinant viruses that were grown on Vero E6 cells.

### 2.2. Cloning of EBOV/Makona Virus Mutants

A modified pSP72 (Promega) cloning vector in which the multiple cloning site (MCS) was replaced by a novel MCS containing AscI and Not I sites (pSPNA) was used to clone the full genomic construct (pSPNA/EBOV-Mak) of EBOV/Makona (Ebola virus/H.sapiens-wt/GIN/2014/Makona-Gueckedou-C07, KJ660347.2). Construct pSPNA/EBOV-Mak was modified to generate a cloned copy of mouse-adapted EBOV/Makona-rgMA (Ebola virus NML/BALB/c-lab/GIN/2014/Makona-Gueckedou-C07-rgMA, MN416402). This plasmid construct, designed for RNA polymerase II based reverse genetics, contained a 5′ cytomegalovirus promoter, hammerhead ribozyme specific for the 5′end of the Makona genome, EBOV/Makona (+sense) genome, β-globin transcription terminator, and Hepatitis delta virus ribozyme 3′ (as described in [21]). To generate the pre-mouse-adapted (preMA) version of EBOV/Makona (EBOV/Makona-preMA), mutations that were associated with the mouse-adaptation of EBOV/Mayinga-MA (AF499101.1) [30] were introduced sequentially into the EBOV/Makona genome by PCR-based mutagenesis (Supplemental Table S1). Mouse adaptation of EBOV/Mayinga-MA resulted in 13 mutations: eight coding mutations, two mutations in the non-coding regions (NCR), and three silent mutations [30]—these mutations are shown in Supplemental Table S2 with the exception of the silent mutations. Of these 13 mutations, two coding mutations were already present in the EBOV/Makona genome (12V in VP35 and 544T in GP). The mutation in the NCR of VP30 was not inserted as it was found not to contribute to virulence of EBOV/Mayinga-MA [30]. To the EBOV/Makona genome, we inserted the following seven mutations: 683 A to G (S72G in NP), 6231 T to C (S65P in GP), 6774 T to C (S246P in GP), 10,342 +A (NCR of VP24), 10,493 C to T (T50I in VP24), 14,380 T to C (F934L in L), and 16,174 A to G (I1532V in L). PrimeSTAR Max (Takara Bio, Mountain View, CA, USA) DNA polymerase was used to introduce each mutation by PCR, and mutated PCR amplicons were inserted back into the full-length EBOV/Makona plasmid by In-Fusion cloning (Takara Bio, Mountain View, CA, USA). The final EBOV/Makona plasmid containing mouse-adapted Mayinga mutations were used for virus rescue by reverse genetics to create the EBOV/Makona-preMA virus.

### 2.3. Virus Rescue and Cells

Viruses were rescued by transfection of GripTite 293 MSR (Life Technologies, Carlsbad, CA, USA) cells with 2 µg of full-length plasmid (pSP-EBOV/Makona-preMA), along with four helper plasmids (kindly provided by Yoshihiro Kawaoka) in the pCAGGS expression plasmid encoding EBOV viral proteins NP (1 µg), VP30 (0.3 µg), VP35 (0.5 µg), and L (1 µg) using 30 µL of TransIT-LT1 (Mirus Bio, Madison, WI, USA) in Opti-MEM (HyClone, GE Healthcare Life Sciences, Logan, Utah, USA), as previously described [21]. Briefly, supernatant was collected 7 days post-transfection and 500 µL of supernatant was blind passaged onto VeroE6 cells (ATCC, Manassas, VA, USA) and monitored for presence of cytopathic effect (CPE) for 14 days. VeroE6 supernatant was collected when 80–90% CPE was observed (EBOV/Makona-preMA P1) and was scaled up to a P2 stock in T150cm^2^ flasks in Dulbecco’s Modified Eagle’s Medium (DMEM, HyClone) supplemented with 1% fetal bovine serum (FBS, HyClone) and 1X L-Glutamine (L-Glu, Gibco, Life Technologies, Grand Island, NY, USA). EBOV viruses were all handled in biosafety level 4 facilities (BSL-4) at the National Microbiology Laboratory of the Public Health Agency of Canada. All experiments described used the P2 stocks of recombinant viruses. The mouse-adapted EBOV/Mayinga that was used for comparison was Ebola virus USAMRIID/BALB/c-lab/COD/1976/Mayinga-MA-p3 (Accession # AF499101.1; EBOV/Mayinga-MA).

### 2.4. Sequencing

Confirmation of viral genome sequences was done by next-generation sequencing. Viral RNA was extracted using the QIAamp Viral RNA Mini Kit (QIAGEN, Hilden, Germany), cDNA was generated with Maxima H minus Double-Stranded cDNA Synthesis Kit (Life Technologies) using the maximum amount of RNA, and dscDNA was directly sequenced in-house using the Illumina MiniSeq platform with library preparation using the Nextera DNA Flex Library Prep Kit (Illumina, San Diego, CA, USA). The reads were trimmed using TrimGalore (version 0.4.4_dev) and host reads were removed by aligning to the African Green monkey genome using bwa (v0.7.17-r1197-dirty). Genomes were assembled using SPAdes (version 3.11.1) [31] with kmer sizes 77, 99, and 127. Contigs containing viral sequences were found using Diamond (version 0.9.22.123) [32] and were compared to a reference EBOV/Makona-Gueckedou-C07 sequence for assembly using nucmer (version 4.0.0beta2) and a custom python script.

The additional mutations that were required to make EBOV/Makona-rgMA were established by sequencing 5 plaque picks of the final lethal mouse-passaged virus. RNA isolation and library preparation were as described above. Reads were cleaned by alignment to the human genome (1000 genomes project, version 37) and mutations were called and analyzed using Breseq version 0.32.1 [33] against a reference EBOV/Makona-Gueckedou-C07 genome sequence. The mutations that were selected were the only ones present in all 5 plaque picks, and a list of all mutations that were identified in each plaque pick is provided in Supplemental Table S3.

### 2.5. Viral Assays

Viral loads were measured by RT-qPCR targeting the EBOV L gene using Light Cycler 480 RNA Master Hydrolysis kit (Roche, Mannheim, Germany), details as previously described [34]. Plaque assay was used to measure viral stock titers. Viral stocks were serially diluted (1:10) in DMEM supplemented with 1% FBS and L-Glu (DMEM/FBS/L-Glu), and 200 µL/well of each dilution was added to a 12-well plate of VeroE6 cells in duplicate. The virus was adsorbed for 1 h at 37°C with 5% CO_2_ and the plates were rocked every 15 min. Inoculum was removed and cells were overlayed with 0.7% SeaPlaque agarose (Lonza, Rockland, ME, USA) in 1X Modified Eagle Medium (MEM, Gibco), 1% FBS, and L-Glu. Plaques were counted 14 days post-infection (dpi). 

Viral titers in blood and tissues were determined by TCID50 assay. Tissue samples were weighed and homogenized in 1 mL of DMEM/1% FBS/L-Glu/PS using a Tissuelyser (QIAGEN) homogenizer. Cell debris was pelleted by centrifugation at 1500 x g for 10 min and supernatant was used for TCID50 assay. Blood and tissue homogenates were serially diluted 1:10, 100 µL of each dilution was added to 96-well plates of VeroE6 cells in triplicate, plates were incubated at 37°C with 5% CO_2_, and the presence of CPE was determined 14 dpi. The TCID50 titer was determined using the Reed and Muench method [35].

### 2.6. Serial Passaging of EBOV/Makona in Suckling Mice

All experiments that used mice were approved by the Animal Care Committee at the Canadian Science Centre for Human and Animal Health in protocol H-17-003. This protocol was carried out in accordance with the guidelines set by the Canadian Council on Animal Care. Mice were euthanized when weight loss ≥ 20% or when severe signs of clinical disease were observed, including paralysis or seizures. 

The starting material for mouse passaging was EBOV/Makona-preMA and EBOV/Makona viruses. Groups of suckling BALB/c mice (*n* = 4 for P1, *n* = 6 for all subsequent passages) were infected via intra-peritoneal (IP) injection of each virus in a volume of 20 µL using the highest available dose to start the P1 passage. For subsequent passaging, livers were collected on day 6 or 7 pi from each group of mice, pooled, and homogenized. Cell debris was pelleted by centrifugation at 1500 x g for 10 min and supernatant was used to infect the next set of naive suckling mice by IP injection with 20 µL of clarified lysate. The procedure was repeated for a total of 7 passages in 3-to-4 day old suckling mice and passage 8 (P8) was in 7-to-8 day old mice. The 9th passage in 14-day old BALB/c mice resulted in lethal infection. Further passaging of EBOV/Makona was not continued once we had generated the virulent strain from the EBOV/Makona-preMA virus. A schematic diagram of the procedure of serial passaging is depicted in Supplemental Figure S1.

### 2.7. Plaque Purification of EBOV/Makona-MA

The P8 mouse-passaged virus was used to grow up a P9 tissue culture stock in VeroE6 cells, and the EBOV/Makona-MA virus from this stock was plaque purified. A standard plaque assay (described in Viral assays) was performed using the P9 tissue culture stock of EBOV/Makona-MA. Using a pipette tip to pierce through the agarose layer, at the location of a plaque that was well separated from other plaques, individual plaque colonies were picked. Each was resuspended in 50 µL of DMEM/FBS/L-Glu and 25 µL was used to inoculate a T25 cm^2^ flask of Vero E6 cells. The remainder was stored at –80°C. Stocks of five individual plaque colonies were grown (EBOV/Makona-MA PP1 to PP5) and sequenced by next-generation sequencing, and based on the consensus mutations found in all 5 clones, 2 additional mutations were consistently acquired during adaptation in mice: 2357 C to A (H630N in NP) and 10979 A to T (K212M in VP24) (Supplemental Table S3). These two mutations were inserted into the EBOV/Makona-preMA genome to generate EBOV/Makona-rgMA. Recombinant viruses containing each mutation, C2357A and A10979T, individually in the background of EBOV/Makona-MA were also constructed and rescued to generate viruses EBOV/Makona-rgMA-2357 and EBOV/Makona-rgMA-10979, respectively.

### 2.8. Characterization of Mouse-Adapted Viruses in BALB/c Mice

In general, the median lethal dose (LD50) was determined by infecting groups of mice (*n* = 3 to 6) with 100 µL IP injection of a dose range of 1 × 10^−3^ to 1 × 10^2^ plaque forming units (PFU) of each virus diluted in MEM + 0.1% bovine serum albumin (BSA, Gibco). For EBOV/Mayinga-MA, mice were infected with a dose range of 4.5 × 10^−3^ to 4.5 x 10^2^ PFU. Mice were monitored for signs of clinical disease and weight loss for up to 18 days and were euthanized when weight loss was ≥20% or when severe signs of clinical disease were observed. LD50 was determined using the Reed and Muench method [36]. 

To further characterize viral replication of mouse-adapted viruses, groups of mice (*n* = 6) were infected IP (100 µL) with 10 PFU of each virus: EBOV/Makona-preMA, EBOV/Makona-rgMA, EBOV/Makona-MA-PP1, EBOV/Makona-MA-2357, EBOV/Makona-MA-10979, and EBOV/Mayinga-MA. Mice were euthanized on day 3 and 5 pi for collection of tissues (liver, kidney, spleen, intestine, lung, brain) and blood for viral titration. Whole blood was collected in K_2_EDTA tubes (BD Microtainer, Franklin Lakes, NJ, USA) for viral titration and complete blood counts, and blood was also collected into serum separator tubes (SST, BD Microtainer) and Lithium Heparin tubes (BD Microtainer) for blood biochemistry analysis. 

### 2.9. Hematology, Blood Biochemistry, and Cytokine Analysis

Whole blood samples were analyzed for complete blood counts using the HM5 hematology system (Abaxis Veterinary Diagnostics). Levels of white blood cells, lymphocytes, monocytes, neutrophils, and platelets were determined. A VetScan VS2 chemistry analyzer (Abaxis Veterinary Diagnostics) along with VetScan Comprehensive Diagnostic Profile reagent rotors (Abaxis, Union City, CA, USA) were used to measure liver and kidney enzymes, glucose, and electrolytes. Serum cytokine responses in mice were examined using the ProcartaPlex Mouse immune Monitoring Panel (48 plex) (Life Technologies). Cytokines that were analysed included: Eotaxin, G-CSF, GM-CSF/CSF-3, Gro-α/KC, IFN-α, IFN-γ, TNF-α, IL-10, IL-12p70, IL-13, IL-17A, IL-18, IL-1α, IL-1β, IL-2, IL-22, IL-23, IL-27, IL-3, IL-31, IL-4, IL-5, IL-6, IL-7, IL-9, IP-10, Leptin, LIF, MCP-1, MCP-3, M-CSF, MIP-1α, MIP-1β, MIP-2, RANTES, sRANKL, VEGF-A, BTC, IL-2RA, IL-7RA, BAFF, IL-28, IL-15/IL-15R, ENA-78 (LIX), IL-25/IL-17, IL-19, ST2/IL-33R, and IL-33. The procedure was as described by the manufacturer’s recommendations, serum samples were diluted 1:5, and test plates were run using a Luminex MAGPIX instrument.

### 2.10. Statistical Analysis

All statistical analyses were performed using GraphPad Prism Software (San Diego, CA, USA). To analyze differences in viral titers, 2-way ANOVA analysis with Bonferroni’s post-tests were performed. For blood counts and biochemistry, 1-way ANOVA analysis with Tukey’s multiple comparison tests were performed. For cytokine analysis, a five-point regression curve was applied for each respective cytokine standard curve, and concentrations of cytokine were interpolated from each respective regression curve. Concentrations were transformed into logarithmic scale before plotting and were analyzed by 2-way ANOVA with Bonferroni’s post-tests. For all analyses, the results were considered statistically significant when a minimum *p*-value <0.05 was attained.

## 3. Results

### 3.1. Generation of EBOV/Makona-MA Virus after Eight Passages in Suckling BALB/c Mice

**Figure 1:**
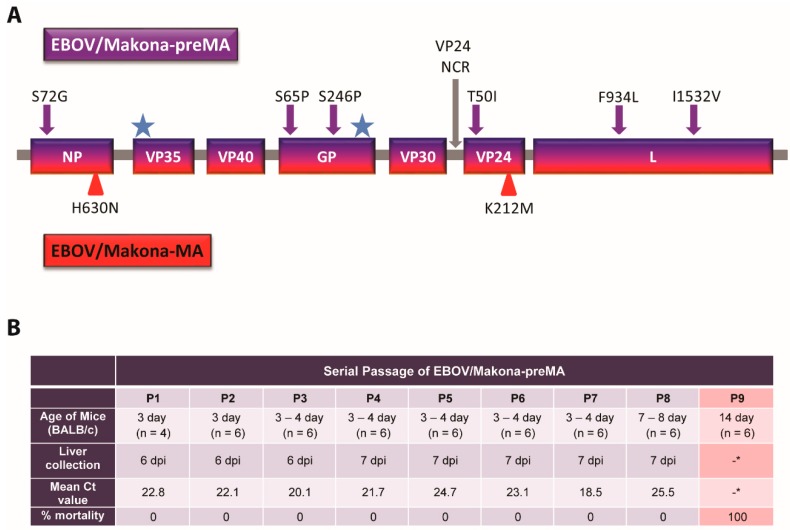
Generation of Ebolavirus (EBOV)/Makona-mouse-adapted (MA) by serial passage in suckling BALB/c mice. (**A**) The EBOV/Makona-preMA genome consisted of six mutations associated with EBOV/Mayinga-MA that were inserted into the coding regions of NP, GP, VP24, and L genes (purple arrows). An additional EBOV/Mayinga-MA mutation in the non-coding region (NCR) of VP24 was inserted (gray arrow). Two EBOV/Mayinga-MA mutations already existing in the EBOV/Makona genome are denoted (blue star). Two additional mutations in NP and VP24 were identified in EBOV/Makona-MA after serial passaging (red triangles). (**B**) Serial passage of EBOV/Makona-preMA in progressively older suckling BALB/c mice resulted in the production of EBOV/Makona-MA. For each passage, livers were collected, pooled, and used to infect a new set of naive mice. Corresponding viral loads were measured by RT-qPCR for EBOV L (mean Ct values shown), *no livers were collected from P9 mice as all mice succumbed to infection.

The EBOV/Makona variant that was isolated during the 2013–2016 West African outbreak shares a high degree of genetic similarity with the EBOV/Mayinga variant that was isolated in 1976 [4]. To generate mouse-adapted EBOV/Makona-MA, we started with the wild-type EBOV/Makona genome and inserted a subset of the mutations identified in EBOV/Mayinga-MA that contribute towards virulence [30]. We hypothesized that prior incorporation of a subset of these mutations into the genome of EBOV/Makona before mouse adaptation might reduce the number of serial passages required to produce EBOV/Makona-MA. Mouse-adaption of EBOV/Mayinga resulted in 13 mutations: eight coding mutations, two mutations in the non-coding regions (NCR), and three silent mutations. In our study, we inserted into the EBOV/Makona genome the following seven mutations that were identified in EBOV/Mayinga-MA: NP (S72G), GP (S65P, S246P), VP24 (+A in NCR, T50I), and L (F934L, I1532V) (Figure 1A). Lastly, the remaining two coding mutations in EBOV/Mayinga-MA were already present in the EBOV/Makona genome (12V in VP35 and 544T in GP), the three silent mutations were not inserted, and the remaining VP30 NCR mutation was not included as it was found not to contribute to the virulence of EBOV/Mayinga-MA [30]. This virus was called EBOV/Makona-preMA and was used as the starting material for mouse adaptation.

Sets of 4 to 6 naïve suckling mice (3–4 days old) were inoculated intra-peritoneally (IP) with EBOV/Makona-preMA, and 6 or 7 days post-infection (dpi), the livers were collected, pooled, and homogenized. The resulting liver homogenate was subsequently administered to the next set of naïve suckling mice and this process was repeated until uniform lethality was observed (Supplemental Figure S1). The presence of viral RNA was detected in the liver homogenates that were collected during each passage by RT-qPCR, and Ct (cycle threshold) values for each passaged virus ranged from 18.5 to 25.5 (Figure 1B). The presence of viral RNA in the liver suggests that viral replication was occurring in the mice during each passage despite a lack of observable clinical signs of disease or death. The eighth passage of the serially passaged virus was uniformly lethal upon IP administration to 14-day-old BALB/c mice. 

It is important to note that we also serially passaged the wild-type EBOV/Makona in mice, and viral loads in the livers had Ct values that ranged from 20.2 to 28.0 (Supplemental Table S4). This data suggests that viral replication was also occurring in these mice. However, after 11 serial passages, EBOV/Makona remained non-lethal in mice and was not further passaged. 

The eighth mouse-passaged (P8) EBOV/Makona-preMA virus was used to generate a P9 stock in Vero E6 cells, representing the fully mouse-adapted virus EBOV/Makona-MA. The P9 stock was plaque purified, and five plaques of EBOV/Makona-MA were grown up as individual stocks (PP1 to PP5). The genome of each of these plaque-picked EBOV/Makona-MA viruses was sequenced to identify mutations that could be virulence determinants in mice. Two nonsynonymous mutations were found consistently in all five plaque picks, 2357 C to A (H630N) in NP and 10,979 A to T (K212M) in VP24, and we hypothesized that these mutations could be important for mouse adaptation of the Makona variant of EBOV (Supplemental Table S3).

### 3.2. Determinants of Virulence in EBOV/Makona-MA

To investigate the determinants of virulence for EBOV/Makona-MA in mice, a reverse genetics (rg) infectious molecular clone of EBOV/Makona-MA, EBOV/Makona-rgMA, was generated by taking the EBOV/Makona-preMA virus and inserting the two mutations that were identified after serial passaging: NP (nt 2357 C to A, H630N) and VP24 (nt 10979 A to T, K212M). In addition to EBOV/Makona-rgMA, we also generated single mutant variants containing either the 2357 (EBOV/Makona-rgMA-2357) or 10,979 (EBOV/Makona-rgMA-10979) mutations, also in the EBOV/Makona-preMA backbone, to study the individual contribution of each mutation to virulence (Supplementary Table S2). The median lethal doses (LD50) for each of these viruses by an IP route were determined in adult BALB/c mice (*n* = 3 to 6), and the LD50 for EBOV/Makona-preMA and EBOV/Mayinga-MA were determined in the same manner for comparison (Table 1); survival and weight loss curves are also provided (Supplemental Figure S2). 

As expected, the EBOV/Makona-preMA was non-lethal at 100 PFU in adult BALB/c mice, which was the highest dose tested. In contrast, the LD50 of EBOV/Makona-rgMA was determined to be 0.004 PFU, a value that is at least 25,000-fold less than the LD50 of EBOV/Makona-preMA virus. The LD50 of EBOV/Mayinga-MA was similar to that of EBOV/Makona-rgMA, having a LD50 value of 0.08 PFU. Mice that were infected with EBOV/Makona-MA viruses all succumbed to infection within 6 to 9 days post-infection (pi), while EBOV/Mayinga-MA infected mice all died around day 5 to 7 pi. Interestingly, EBOV/Makona-rgMA-10979 had the same LD50 value of 0.004 PFU as EBOV/Makona-rgMA, while EBOV/Makona-rgMA-2357 remained non-lethal at all the doses that were tested. These results suggest that nt 10979 mutation (A to T) in VP24 is a critical virulence determinant of EBOV/Makona-rgMA in mice, while the nt 2357 mutation (C to A) in NP does not significantly contribute to pathogenicity.

### 3.3. EBOV/Makona-MA Virus Replicates to High Titers in Mice

To further characterize the four EBOV/Makona-MA variants in vivo, a serial sacrifice study was carried out in adult mice to compare the viral burden in the blood and tissues following exposure to EBOV/Makona-rgMA, EBOV/Makona-rgMA-10979, EBOV/Makona-rgMA-2357, EBOV/Makona-preMA, and EBOV/Mayinga-MA. In addition, one of the plaque purified viruses of EBOV/Makona-MA (PP1) was included to confirm that the reverse genetics-generated version of EBOV/Makona-MA behaved similarly to the viruses that were isolated directly from the mice. For this experiment, mice were infected by IP injection with 10 PFU of each virus. On days 3 and 5 pi, mice from each group were euthanized and blood, serum, and tissues were collected for analysis. 

**Figure 2:**
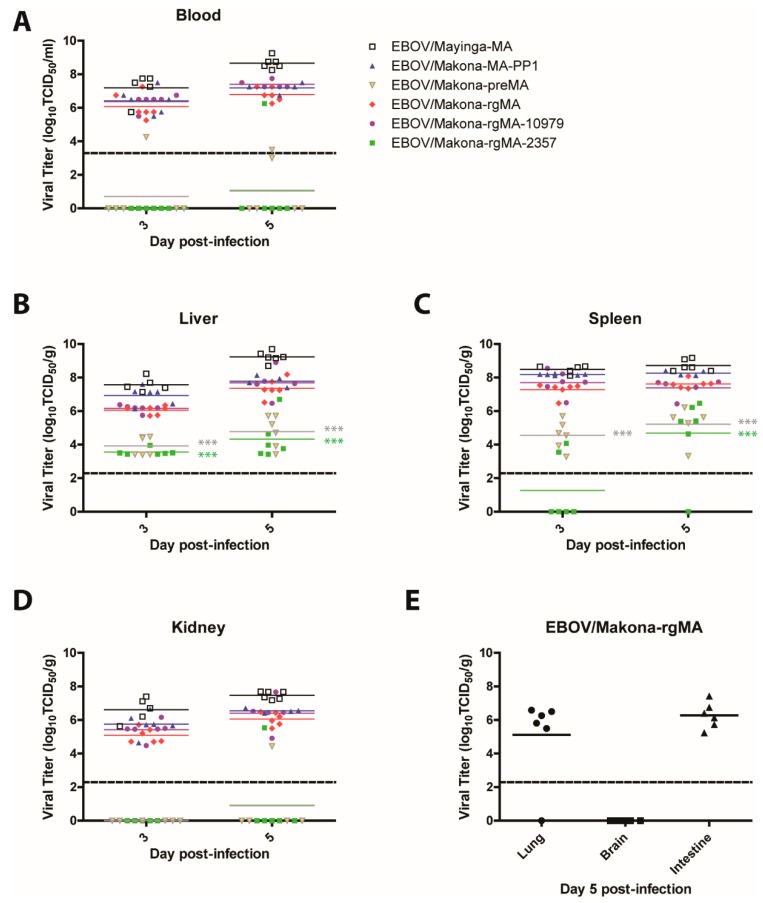
Viremia and viral replication in tissues of mice that were infected with mouse-adapted EBOV. Groups of mice (*n* = 6) were infected intraperitoneally with 10 PFU of either EBOV/Mayinga-MA, EBOV/Makona-MA-PP1, EBOV/Makona-preMA, EBOV/Makona-rgMA, or the two single mutant viruses EBOV/Makona-rgMA-10979 or EBOV/Makona-rgMA-2357. (**A**) Blood, (**B**) liver, (**C**) spleen, and (**D**) kidney samples were collected on days 3 and 5 post-infection. (**E**) Viral dissemination to the lung, brain, and intestine was also examined for EBOV/Makona-rgMA infected mice on day 5 post-infection. Mean viral titers are shown, 2-way ANOVA with Bonferroni post-tests was performed, *** *p*-value < 0.001. The dashed line denotes the limit of detection of the TCID50/mL or TCID50/g assay.

Levels of viremia were the highest in the mice that were infected with EBOV/Mayinga-MA, EBOV/Makona-MA-PP1, EBOV/Makona-rgMA, and EBOV/Makona-rgMA-10979. All of these viruses showed similarly high levels of viremia, with mean titers ranging from 6.8 to 8.7-log TCID50/mL on day 5 pi. In contrast, mice that were infected with EBOV/Makona-preMA or EBOV/Makona-rgMA-2357 viruses had mean viral titers that were below the limit of detection on both days (Figure 2A). Correspondingly, measurement of viral replication in the tissues (liver, spleen, and kidney) revealed a similar trend where the highest level of virus was present in mice that were infected with EBOV/Mayinga-MA, EBOV/Makona-MA-PP1, EBOV/Makona-rgMA, and EBOV/Makona-rgMA-10979 (Figure 2B–D). Although liver viral titers reaching between 3.6 to 4.8-log TCID50/mL were measured in mice that were infected with either EBOV/Makona-preMA or EBOV/Makona-rgMA-2357, these values were significantly lower compared to EBOV/Makona-rgMA on both days (*p*-values < 0.001). Interestingly, on day 3 pi, in the spleen, there were only two mice with detectable EBOV/Makona-rgMA-2357 compared to a mean titer of 4.6-log TCID50/mL that was found in EBOV/Makona-preMA-infected mice (Figure 2C), suggesting that EBOV/Makona-rgMA-2357 viral spread to the spleen may be delayed. In the kidney, mean viral titers for EBOV/Makona-preMA- and EBOV/Makona-rgMA-2357-infected mice were below the limit of detection (Figure 2D). To further investigate the dissemination of EBOV/Makona-rgMA in mice, we also measured the viral titers in the lung, brain, and intestine. Surprisingly, no virus could be detected in the brain of these mice, while mean titers of 5.1 and 6.3-log TCID50/mL were detected in the lung and intestine, respectively (Figure 2E). These results corresponded with the LD50 values for each virus, where EBOV/Makona-preMA and EBOV/Makona-rgMA-2357 remained non-lethal even at the highest dose tested, while EBOV/Makona-rgMA, EBOV/Makona-rgMA-10979, and EBOV/Mayinga-MA all had very low LD50 values. This data confirms the importance of the mutation at 10,979 in VP24 of EBOV/Makona-MA for virulence, while the 2357 mutation in NP is not required. Additionally, the reverse genetics version of EBOV/Makona-MA (EBOV/Makona-rgMA) replicated to a similar level as the original mouse-isolated virus EBOV/Makona-MA-PP1. 

### 3.4. EBOV/Makona-MA Infection in Mice Induces Lymphopenia and Neutrophilia

Blood samples that were collected from mice infected with 10 PFU of each virus (EBOV/Makona-rgMA, EBOV/Makona-rgMA-10979, EBOV/Makona-rgMA-2357, EBOV/Makona-preMA, EBOV/Makona- MA-PP1, and EBOV/Mayinga-MA) were also analyzed for changes in hematology and blood biochemistry on days 3 and 5 pi. As controls, blood from a group of uninfected mice (*n* = 6) was collected on day 0 to indicate baseline levels for each test. It is important to note that as the mice progressed to severe disease by day 5 pi, some mice succumbed to infection before they could be sampled, while others became difficult to bleed under anesthesia. This resulted in fewer sample values (*n* = 1 to 6) from mice that were infected with more virulent viruses such as EBOV/Mayinga-MA, EBOV/Makona-MA-PP1, EBOV/Makona-rgMA-10979, and EBOV/Makona-rgMA. 

**Figure 3:**
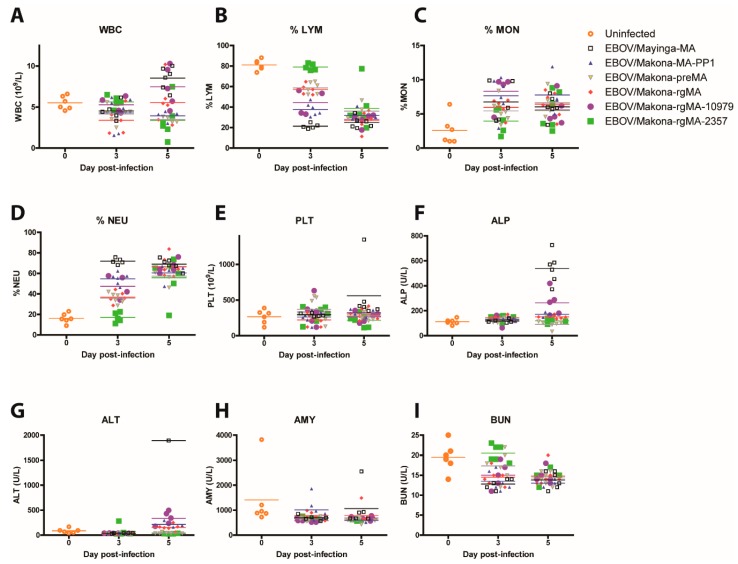
Hematology and blood biochemistry in mice who were infected with MA-EBOV. Groups of mice (*n* = 6) were infected intraperitoneally with 10 PFU of either EBOV/Mayinga-MA, EBOV/Makona-MA-PP1, EBOV/Makona-preMA, EBOV/Makona-rgMA, or the two single mutant viruses EBOV/Makona-rgMA-10979 or EBOV/Makona-rgMA-2357. Blood samples were collected on days 3 and 5 post-infection and (**A**) total white blood cell count (WBC), (**B**) % lymphocytes (%LYM), (**C**) % monocytes (%MON), (**D**) % neutrophils (%NEU), (**E**) platelets (PLT), (**F**) alkaline phosphatase (ALP), (**G**) alanine aminotransferase (ALT), (**H**) amylase (AMY), and (**I**) blood urea nitrogen (BUN) were measured. Blood was also collected on day 0 from a group of uninfected BALB/c mice (*n* = 6) to establish baseline levels for all the parameters.

Total white blood cell (WBC) counts were determined and all mean values showed minimal differences compared to each other and to the mock samples on day 3 pi, with the exception of EBOV/Mayinga-MA and EBOV/Makona-rgMA-10979 on day 5 pi, which had the highest mean counts of 8.5 and 7.5 10^9^/L, respectively, while EBOV/Makona-rgMA-2357 had the lowest mean value of 3.4 10^9^/L (Figure 3A). On day 3 pi, total % lymphocytes (%LYM) were significantly decreased by ~20–60% for all groups in comparison to EBOV/Makona-rgMA-2357, which had a mean value of 80% LYM (*p*-values < 0.001 to 0.01), which declined to similar levels to all the other groups by day 5 pi (~25–40% LYM) (Figure 3B). As levels of lymphocytes declined, we saw a corresponding increase in the levels of monocytes (MON) and neutrophils (NEU) on day 5 pi (Figure 3C,D). On day 3 pi, EBOV/Makona-rgMA-2357 infected mice had ~17% NEU, which was significantly lower compared to all other groups by ~18–55% (*p*-values < 0.001 to 0.01), and by day 5 pi, all groups had similar values ranging from ~55% to 70% NEU. No changes were observed in platelet (PLT) counts for all groups on either day (Figure 3E). Lymphopenia is a typical clinical manifestation of EVD that is also observed in EBOV-infected non-human primates (NHPs). The observation of lymphopenia in EBOV/Makona-rgMA-infected mice that coincided with EBOV/Mayinga-MA-infected mice shows the ability of EBOV/Makona-rgMA to recapitulate characteristics of EVD in mice.

As EVD progresses in animals, liver and kidney functions generally decrease and changes in enzymes that are associated with organ function can be measured as an indicator of the severity of the disease caused by EBOV infection. Levels of alkaline phosphatase (ALP) and alanine aminotransferase (ALT) were measured as indicators of liver disease, amylase (AMY) for kidney and pancreatic disease, and blood urea nitrogen (BUN) for both liver and kidney diseases. Minimal changes to ALP and ALT levels were observed for all groups with the exception of EBOV/Mayinga-MA, which had significantly higher ALP levels of ~540 U/L on day 5 pi compared to all other groups (*p*-value < 0.001) (Figure 3F). The one mouse that we could determine ALT levels for EBOV/Mayinga-MA had 1893 U/L, which was about 5-fold higher compared to all the other groups (Figure 3G). No differences were observed for AMY between all the groups, while a significant decrease in BUN was detected for all groups compared to EBOV/Makona-rgMA-2357 on day 3 pi (*p*-value < 0.001 to 0.05) (Figure 3H,I). However, by day 5 pi, the levels of BUN decreased for EBOV/Makona-rgMA-2357 to similar levels to all the other groups. Despite high viral titers on day 5 pi, for the majority of viral groups that were tested, except EBOV/Makona-preMA and EBOV/Makona-rgMA-2357, signs of liver dysfunction were not observed, which suggests that day 5 may be too early for these responses to be seen.

### 3.5. Cytokine and Chemokine Responses in Mice Infected with EBOV/Makona-MA

Excessive activation of pro-inflammatory cytokines and chemokines is a hallmark of EVD, which can lead to tissue damage and contribute to death. To examine whether EBOV/Makona-rgMA induces a pro-inflammatory response in infected mice that is consistent with that observed with EBOV/Mayinga-MA, differences in cytokine and chemokine expression in the serum of mice that were infected with EBOV/Makona-rgMA, EBOV/Makona-rgMA-10979, EBOV/Makona-rgMA-2357, EBOV/Makona-preMA, EBOV/Makona-MA-PP1, and EBOV/Mayinga-MA were studied. To determine baseline levels of each cytokine or chemokine, serum from a group of uninfected BALB/c mice (*n* = 6) was also analyzed for comparison. Although the majority of the 48 cytokines and chemokines tested showed no significant differences between all the virus groups, there were some notable differences in proteins that were associated with pro-inflammatory and antiviral responses.

**Figure 4:**
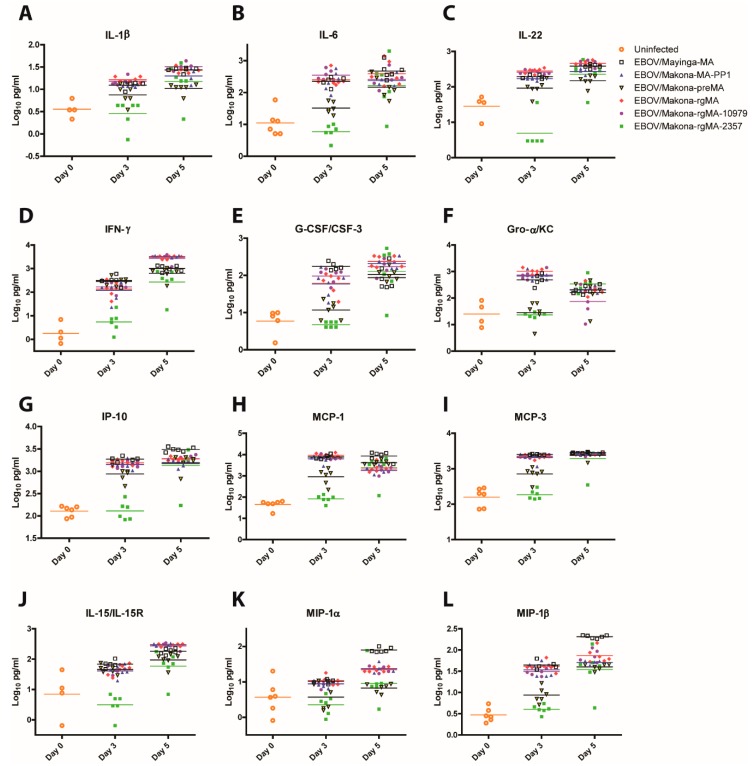
Cytokine and chemokine responses in mice that were infected with MA-EBOV. Groups of mice (*n* = 6) were infected intraperitoneally with 10 PFU of either EBOV/Mayinga-MA, EBOV/Makona-MA-PP1, EBOV/Makona-preMA, EBOV/Makona-rgMA, or the two single mutant viruses EBOV/Makona-rgMA-10979 or EBOV/Makona-rgMA-2357. Serum samples were collected on days 3 and 5 post-infection and expression levels of (**A**) IL1-β, (**B**) IL-6, (**C**) IL-22, (**D**), IFN-γ, (**E**) G-CSF/CSF-3, (**F**) Gro-α/KC, (**G**) IP-10, (**H**) MCP-1, (**I**) MCP-3, (**J**) IL-15/IL-15R, (**K**) MIP1-α, and (**L**) MIP-1β were determined by Luminex assay.

In general, viruses with the greatest viremia and replication in organs (EBOV/Makona-rgMA, EBOV/Makona-MA-PP1, EBOV/Makona-rgMA-10979, and EBOV/Mayinga-MA) induced a strong pro-inflammatory (IL-1β, IL-6, and IL-22) and type II interferon response (IFN-γ) (Figure 4A–D). Interestingly, EBOV/Makona-preMA-infected mice that had minimal viremia and reduced viral replication in the liver and spleen expressed similarly high levels of pro-inflammatory cytokines and IFN-γ with the exception of IL-6, where its mean value of 1.5 log_10_pg/mL was approximately 40-fold less than the more pathogenic viruses (Figure 4B). In contrast, on day 3 pi, EBOV/Makona-rgMA-2357-infected mice had significantly lower expression of pro-inflammatory cytokines and IFN-γ compared to all the other virus groups (*p*-value < 0.001 to 0.01). However, these differences were not observed on day 5 pi as levels of these cytokines increased to similar levels as all the other groups. 

A similar trend in the expression of chemokines that attracts leukocytes including neutrophils, monocytes/macrophages, NK, and DC cells to sites of inflammation was observed. The viruses that replicate well in mice had the greatest expression levels of G-CSF/CSF-3, Gro-α/KC, IP-10, MCP-1, MCP-3, IL-15/IL-15R, MIP-1α, and MIP-1β (Figure 4E–L). Interestingly, EBOV/Makona-preMA- and EBOV/Makona-rgMA-2357-infected mice expressed similarly reduced levels of G-CSF/CSF-3, Gro-α/KC, and MIP-1α and MIP-1β. Although, for other chemokines, EBOV/Makona-preMA-infected mice expressed levels of MCP-1 and MCP-3 that were higher than EBOV/Makona-rgMA-2357-infected mice and levels of IP-10 and IL-15/IL-15R that were similar to levels that were expressed by the more virulent viruses. 

Overall, these results show that virulent viruses that replicate well in mice, such as EBOV/Makona-rgMA, EBOV/Makona-MA-PP1, EBOV/Makona-rgMA-10979, and EBOV/Mayinga-MA, induce strong pro-inflammatory and type II IFN responses. Furthermore, these viruses are able to recruit more target cells such as monocytes/macrophages and DC cells to sites of infection through increased secretion of specific chemokines. Strong activation of pro-inflammatory responses are also observed in EBOV-infected NHPs, which are considered the gold standard animal model for recapitulating human EVD. Furthermore, the ability of EBOV/Makona-rgMA to induce pro-inflammatory responses that are similar to EBOV/Mayinga-MA highlights the applicability of using EBOV/Makona-rgMA-infected mice as a good model of EVD. Interestingly, differences in cytokine and chemokine expression existed between EBOV/Makona-preMA and EBOV/Makona-rgMA-2357 despite their shared replication deficiency in mice, suggesting that the additional 2357 mutation in NP may play a role in modulating the host response to infection.

## 4. Discussion

We have developed a new mouse model of EBOV infection based on an isolate from the 2014 West African outbreak, EBOV/Makona-MA. This mouse-adapted virus was generated after eight passages in progressively older suckling BALB/c mice and was compared to nine passages that were required to generate EBOV/Mayinga-MA almost 20 years ago [22], by starting with a genetically engineered virus that contained the amino acid changes that were associated with mouse-adaptation from the EBOV/Mayinga-MA virus. Although Bray et al. [22] were able to generate a mouse adapted strain in only nine passages, starting from a virus that had been passaged in brains of suckling mice three times, we believe that we likely produced a mouse-adapted version of the virus faster than the conventional method of passaging the wild-type virus through the same animal host. In recent adaptations of filoviruses to rodent hosts, it took 24 passages to adapt the Angola variant of Marburg virus to mice [28], 25 passages to adapt Sudan virus to guinea pigs [37], and 24 serial passages in both SCID and BALB/c mice were needed to generate a lethal Ravn virus strain [29]. On the other hand, only nine host passages were required to adapt the Marburg virus to guinea pigs [38]. Since EBOV/Makona remained non-lethal after 11 passages in mice, this suggests that the introduction of the EBOV/Mayinga-MA mutations assisted the adaptation process, though it is likely that continued passaging of EBOV/Makona in mice would eventually result in a mouse-adapted virus. Our method of inserting mutations to stimulate rapid mouse-adaptation may be a useful approach that may be applied to future Ebola viruses with high sequence identity in the regions where these mutations are found. Further investigation of the mechanism by which these mutations contribute to virulence may help provide insight into conserved viral protein functions that are important for virulence.

Sequencing of EBOV/Makona-MA revealed two additional mutations after passaging, in NP (nucleotide 2357, H630N) and VP24 (nucleotide 10979, K212M). From our results, it was clear that the mutation in VP24 was critical for pathogenicity since EBOV/Makona-rgMA-10979 behaved similarly to EBOV/Makona-rgMA, EBOV/Makona-MA-PP1, and EBOV/Mayinga-MA in terms of uncontrolled viral replication, increased release of pro-inflammatory cytokines and chemokines, lymphopenia, and the ability to cause severe disease and lethal infection in mice. Interestingly, the VP24 mutation in EBOV/Mayinga-MA was also identified as one of the key determinants of virulence based on its role as an IFN antagonist [30]. VP24 blocks IFN-α/β/γ signaling through its interaction with the NPI-1 subfamily of karyopherin alpha nuclear localization signal receptors that are needed for nuclear importation of phosphorylated STAT1 proteins [39,40,41]. By preventing phosphorylated STAT from entering the nucleus, subsequent activation of IFN-stimulated genes does not occur, impairing the host antiviral response [41]. 

Interestingly, in the VP24 gene of EBOV/Mayinga-MA, a methionine is present at residue 212. This suggests that adaptation of EBOV/Makona-MA required this K212M mutation to acquire the same amino acid identity that is already present in EBOV/Mayinga-MA, emphasizing the importance of this residue for virulence. This mutation was not found during passaging of EBOV/Mayinga as the original isolate that was used for adaptation already contained M212 [22]. The complete genome sequences of six other isolates of EBOV in 1976 also all contain methionine at residue 212 in the VP24 gene. In comparison, isolates of EBOV after 1976 including viruses from 1995 [KU182909], 2002 [KC242800], and 2018 [MK007344] all contain K212, similar to EBOV/Makona. Therefore, the EBOV/Mayinga isolate containing M212 in VP24 may have already had an advantage towards mouse-adaptation. Had an EBOV isolate from later than 1976 been chosen for mouse-adaptation, the number of passages required for full adaptation may have taken longer due to the presence of K212 in VP24. The finding that original EBOV isolates from 1976 contained a M212 in VP24 that is changed to a K212 in more recent isolates, coupled with the importance of this residue for mouse adaptation, is interesting and emphasizes the importance of further studying the role of this residue for virulence. 

In contrast, the inability of EBOV/Makona-rgMA-2357 to cause lethal disease, viremia, and replication in tissues shows that this mutation does not contribute to virulence in mice. Not surprisingly, this lack of virulence was also observed in EBOV/Makona-preMA infected mice. The only difference between the two viruses is the presence of the 2357 mutation (H630N in NP) in EBOV/Makona-rgMA-2357. NP plays an important role in viral RNA encapsidation and formation of a RNA-dependent RNA polymerase (RDRP) complex that is needed for viral transcription and replication [42]. The detection of EBOV/Makona-rgMA-2357 titers in the livers of infected mice, along with the fact that this mutation was also present in EBOV/Makona-rgMA and EBOV/Makona-MA-PP1 viruses that replicated to high titers, suggests that the NP mutation does not interfere with viral replication. Overall, this extra NP mutation most likely appeared alongside the VP24 mutation during mouse passaging and is not required for virulence, although it does have a minor effect on host innate immune response based on specific cytokine/chemokine expression differences observed at day 3 pi compared to EBOV/Makona-preMA.

Primary target cells of filovirus infection are monocytes/macrophages and dendritic cells [43,44,45,46]. Infection of these migrating cells helps spread the virus from infection sites to other target organs like the liver and spleen. Excessive activation of the pro-inflammatory response is another hallmark of EVD, resulting in tissue damage, multi-organ failure, and eventual death [46,47]. In our model, we saw significant increases in levels of pro-inflammatory cytokines and chemokines associated with the recruitment of EBOV target cells and lymphopenia in EBOV/Makona-MA infected mice. These clinical manifestations of disease that are caused by EBOV/Makona-MA infection in mice are consistent with those observed in EBOV infected non-human primates (NHPs), which are considered the gold standard animal model for recapitulating human EVD disease most accurately [45,47,48].

The Mayinga and Makona variants of EBOV were isolated almost 40 years apart, yet they share ~97% sequence identity [4]. The fact that EBOV/Makona-MA VP24 gene acquired a K212M mutation that changed the sequence to that originally found in EBOV/Mayinga-MA emphasizes the critical role of this residue for virulence, which may be to facilitate its function as an IFN-antagonist in mice. Due to the absolute requirement of this mutation for mouse-adaptation of EBOV/Makona, this mutation should be included in the list of known mutations that are important for EBOV mouse-adaption to use for future generation of MA-EBOV variants. Although these two viruses are closely related, differences in pathogenicity between these viruses in animal models have been noted, where EBOV/Makona shows reduced pathogenicity compared to EBOV/Mayinga [49]. The approximately 8500 EVD survivors from the 2013–2016 West African outbreak and the rise in EBOV persistence stemming from this large cohort of survivors [6,7,8,9,10] highlights the importance of having an animal model to study persistent infections in immunoprivileged sites, and possibly by studying EBOV/Makona-rgMA-infected mice, this may help shed more light on this topic. Although mouse models recapitulate human and NHP EVD well, the presence of coagulopathy, rash, and hemorrhagic manifestations are not observed in mice [23], which may limit the extrapolation of therapeutic protective efficacies in mice to other animal models. However, small rodent models are still desirable as an initial step to evaluate vaccines and therapeutics because they are less sentient, reagents are easily accessible, and transgenic mice are an option. Our method to rapidly generate a mouse-model of EVD may prove valuable as new isolates of EBOV emerge, and updated animal models are needed to evaluate effective countermeasures for those circulating variants of the virus. 

## Figures and Tables

**Table 1:** Virulence comparison of mouse-adapted EBOV/Makona viruses to mouse-adapted EBOV-Mayinga. Groups of 4 to 6 week old BALB/c mice (*n* = 3 to 6) were infected by intraperitoneal injection with either EBOV/Makona-preMA, reverse genetics generated EBOV/Makona-MA (EBOV/Makona-rgMA), EBOV/Makona-MA with either the 2357 mutation (EBOV/Makona-rgMA-2357) or the 10,979 mutation (EBOV/Makona-rgMA-10979), or EBOV/Mayinga-MA with various doses shown in plaque forming units (PFU). Survival and weight loss were monitored for up to 18 days post-infection. Median lethal dose (LD50) was calculated using the Reed and Muench method.

**Virus**	**Survival at Dose Tested (PFU)**						**LD_50_ (PFU)**
	**0.001**	**0.01**	**0.1**	**1**	**10**	**100**	
**EBOV/Makona-preMA**						6/6	>100
**EBOV/Makona-rgMA**	6/6	1/6	2/6	0/6	0/3		0.004
**EBOV/Makona-rgMA-2357**			3/3	3/3	3/3	3/3	>100
**EBOV/Makona-rgMA-10979**	6/6	1/6	1/6	0/6	0/6	0/3	0.004
	**PFU**						
	**0.0045**	**0.045**	**0.45**	**4.5**	**45**	**450**	
**EBOV/Mayinga-MA**	2/3	2/3	0/3	1/3	0/3	0/3	0.08

## Supplementary Materials

The following are available online at https://www.mdpi.com/1999-4915/11/11/987/s1, Figure S1: Generation of mouse-adapted EBOV/Makona by serial passaging in suckling BALB/c mice, Figure S2: Survival and weight loss curves for mouse-adapted Ebola viruses, Table S1: List of mutagenesis primers used to create EBOV/Makona-preMA and EBOV/Makona-rgMA, Table S2: Comparison of amino acid identities in the genes of human and mouse-adapted Ebola viruses (EBOV), Table S3: Next-generation sequencing of EBOV/Makona-MA plaque picks 1 to 5, Table S4: Serial passage of wild-type EBOV/Makona in suckling BALB/c mice.
