# Supplementary material for: Generation and Characterization of a Mouse-Adapted Makona Variant of Ebola Virus
Source: Viruses. 2019 Oct 26;11(11):987. doi: 10.3390/v11110987 (PMC6893688; doi:10.3390/v11110987)
Supplement: Supplementary file 1 [file viruses-11-00987-s001.pdf]

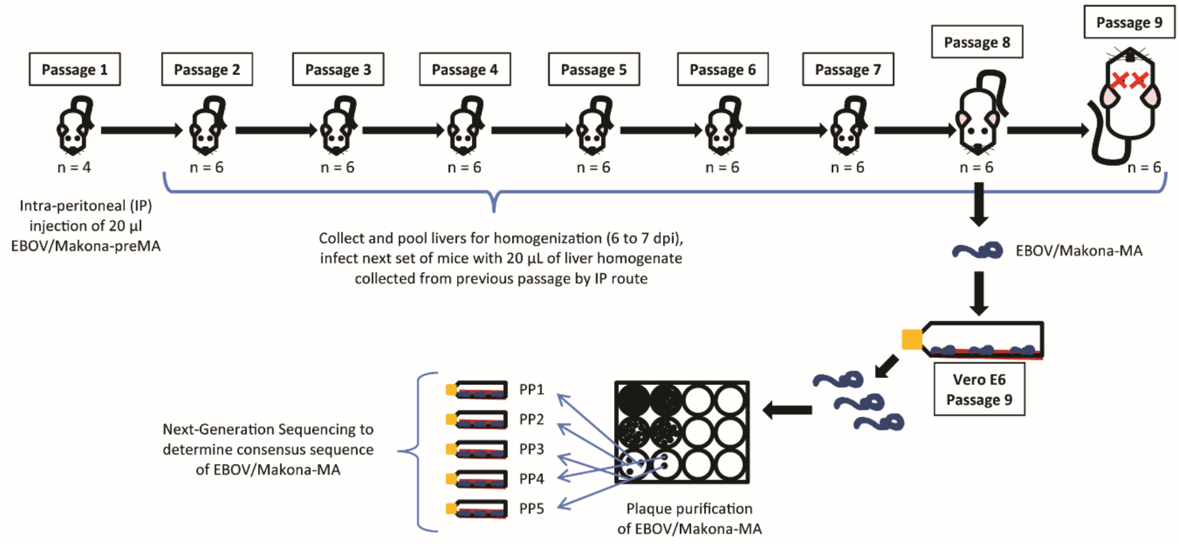

Supplemental Figure S1. Generation of mouse-adapted EBOV/Makona by serial passaging in suckling BALB/c mice. For passage 1, mice (*n* = 4, 3-to-4 days old) were infected by intra-peritoneal (IP) injection with 20 µl of EBOV/Makona-preMA. Livers were collected from the mice on day 6 or 7 post-infection (dpi) and were pooled, homogenized, and clarified of cell debris by centrifugation. For passage 2, liver homogenate that was collected from the previous passage was used to infect a new set of naïve suckling mice (*n* = 6, 3-to-4 days old). This process was repeated for a total of 7 passages in 3-to-4 day old suckling mice, while passage 8 was used to infect 7-to-8 day old suckling mice. The 9th passage in 14-day old mice resulted in uniform lethality. The passage 8 liver homogenate was used to grow a tissue culture stock using Vero E6 cells. The tissue culture stock of EBOV/Makona-MA was plaque purified by standard plaque assay, and five individual plaques were chosen for subsequent stock generation (PP1 to PP5). Viral RNA was extracted from each of these stocks, and viral genomes were sequenced by Next-generation Sequencing to determine the consensus sequence of EBOV/Makona-MA.

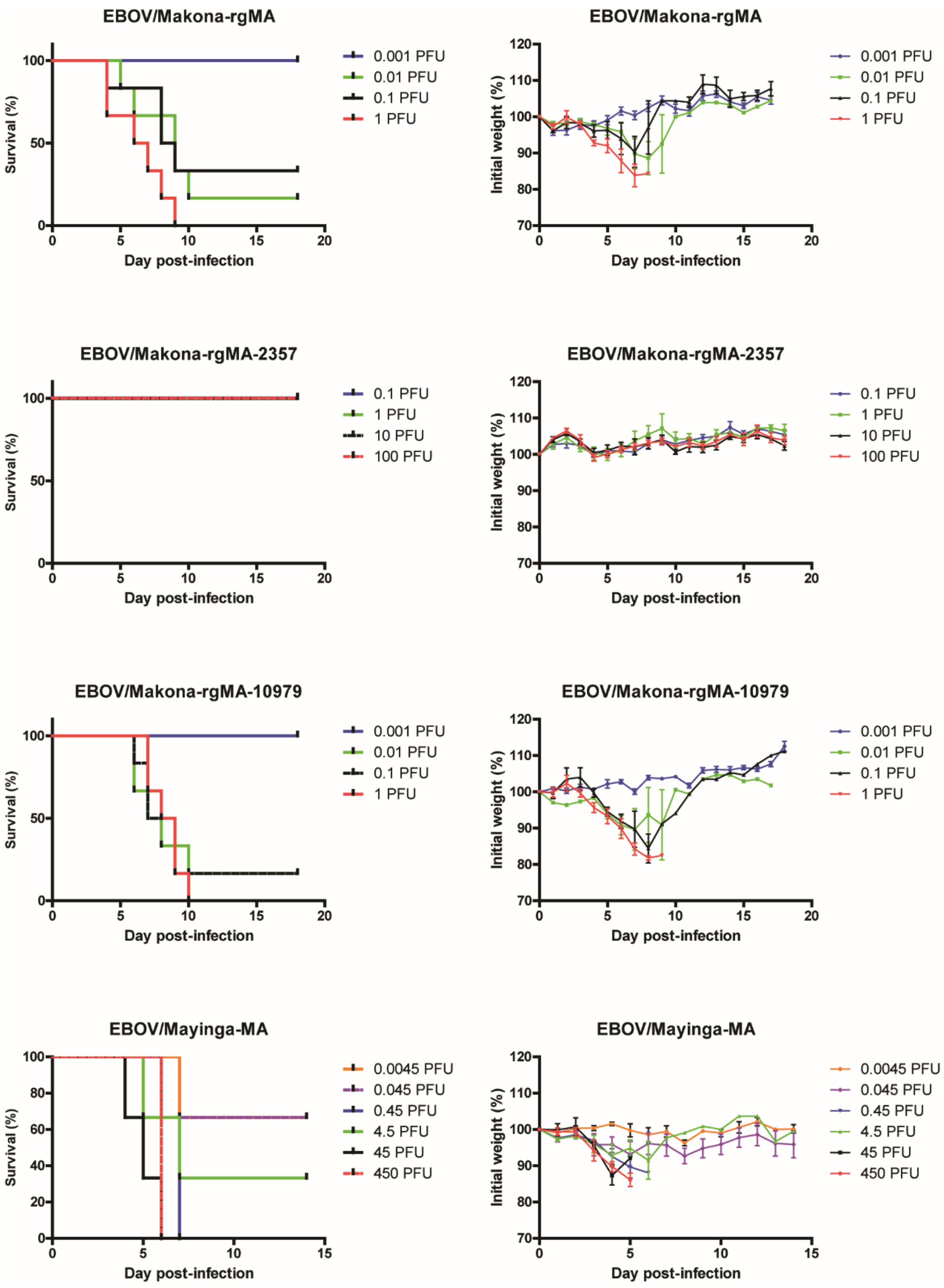

Supplemental Figure S2. Survival and weight loss curves for mouse-adapted Ebola viruses. Groups of BALB/c mice (*n* = 3 to 6) were infected by intra-peritoneal injection with either EBOV/Makona-rgMA, EBOV/Makona-rgMA-2357, EBOV/Makona-rgMA-10979, or EBOV/Mayinga-MA. Ten-fold serial dilutions of viral doses were performed and administered to each group. Survival and weight loss were monitored for 14–18 days post-infection.

Supplemental Table S1. List of mutagenesis primers used to create Ebolavirus (EBOV)/Makona-preMA (mouse-adapted) and EBOV/Makona-rgMA. Nucleotides that are highlighted in red indicate the mutation site.

Mutagenesis Primers	
C07-MA-678F	CGGACGGTTTCCTTCTCATGCTTTGTC
C07-MA-688R	GAAACCGTCCGCACTCTCTTGAAAATC
C07-MA-6226F	TGAGACCAGTTGGACTGAATCTCGAG
C07-MA-6236R	AACTGGTCTCAATTGATTTGTGGATGAC
C07-MA-6769F	TTGAACCAAGATTCACACCACAGTTTC
C07-MA-6779R	TCTTGGTTCAAGTTGGACGTAGGTCAA
C07-MA-10338F	AAAAAACCATGGCCAAAGCTACGGG
C07-MA-10348R	CATGGTTTTTTTCTCAGGTCTTGCTTGG
C07-MA-10489F	TGTGATTCACAAAGGAATGGCCCTATTGC
C07-MA-10499R	TGTGAATCACATCAAACTCAATACCAGC
C07-MA-14376F	GTTTACTCCAGTTAAAAACTTATCTCCG
C07-MA-14386R	CTGGAGTAAACCTGAGGTAACTGGATC
C07-MA-16170F	TTTCTGTTTACATAGGCGGTGCTGCA
C07-MA-16180R	GTAAACAGAAAAATGGGGATCAATACTC
C07-MA-2357F	AGATCAGGACAACATTCAAGAG
C07-MA-2357R	CTCTTGAATGTTGTCCTGATCT
C07-MA-10979F	ATGAACCGCATGAAGCCTGG
C07-MA-10979R	CCAGGCTTCATGCGGTTCAT

Supplementary Table S2. Comparison of amino acid identities in the genes of human and mouse-adapted Ebola viruses (EBOV). EBOV/Mayinga is a human isolate of Ebola virus from 1976, a mouse-adapted version of the virus was generated, EBOV/Mayinga-MA, and the differences in amino acid sequences in each protein are shown (highlighted in blue). EBOV/Makona is a human isolate from 2014, EBOV/Makona-preMA contains a subset of mutations found in EBOV/Mayinga-MA, and was used as the starting virus for passaging in mice to generate the full mouse-adapted version of EBOV/Makona. EBOV/Makona-rgMA is the reverse genetics generated mouse-adapted EBOV/Makona, containing an amino acid change in NP (highlighted in red) and VP24 (highlighted in yellow), which differs from EBOV/Makona-preMA. Two variants of EBOV/Makona-rgMA were generated, EBOV/Makona-rgMA-2357 and EBOV/Makona-rgMA10979.

Ebola Virus	NP		VP35	GP			VP30	VP24			L	
EBOV/Mayinga	S72	H630	12A	S65	S246	544I	NCR nt 9563A	NCR	T50	M212	F934	I1532
EBOV/Mayinga-MA	G72	H630	12V	P65	P246	544T	NCR nt 9563G	NCR nt 10343+A	I50	M212	L934	V1532
EBOV/Makona	S72	H630	12V	S65	S246	544T	NCR nt 9563A	NCR	T50	K212	F934	I1532
EBOV/Makona-preMA	G72	H630	12V	P65	P246	544T	NCR nt 9563A	NCR nt 10343+A	I50	K212	L934	V1532
EBOV/Makona-rgMA	G72	N630	12V	P65	P246	544T	NCR nt 9563A	NCR nt 10343+A	I50	M212	L934	V1532
EBOV/Makona-rgMA-2357	G72	N630	12V	P65	P246	544T	NCR nt 9563A	NCR nt 10343+A	I50	K212	L934	V1532
EBOV/Makona-rgMA-10979	G72	H630	12V	P65	P246	544T	NCR nt 9563A	NCR nt 10343+A	I50	M212	L934	V1532

Supplemental Table S3. Next-generation sequencing of EBOV/Makona-MA plaque picks 1 to 5. A virus stock of EBOV/Makona-MA passaged eight times in mice was generated in Vero E6 cells using liver homogenate that was collected from mice. This tissue culture stock of EBOV/Makona-MA was plaque purified by standard plaque assay, and five individual plaques (PP1 to PP5) were chosen and stocks of each plaque were grown up using Vero E6 cells. Viral RNA from each EBOV/Makona-MA-PP1 to PP5 stocks was extracted and next-generation sequencing was performed using the Illumina MiniSeq platform with library preparation using the Nextera DNA Flex Library Prep Kit. Viral sequences obtained were compared to a reference EBOV/Makona-Gueckedou-C07 sequence for assembly. The percent frequency of mutations in each plaque pick are shown.

	Position	Mutation	Freq %	Amino Acid	Gene
EBOV/Makona-MA-PP1	683	A → G	100	S72G	NP
	2357	C → A	100	H630N	NP
	3012	A → G	100		NP/VP35 intergenic region
	5684	T → G	100		VP40/GP intergenic region
	6231	T → C	100	S65P	GP
	6774	T → C	100	S246P	GP
	8021	A → G	5.7	I367V	GP
	9849	C → T	100		VP30/VP24 intergenic region
	10342	$(A)_{6\rightarrow 7}$	100		VP30/VP24 intergenic region
	10493	C → T	100	T50I	VP24
	10979	A → T	100	K212M	VP24
	14380	T → C	100	F934L	L
	16174	A → G	100	I1532V	L
	18813	T → C	100		L /- intergenic region
EBOV/Makona-MA-PP2	683	A → G	100	S72G	NP
	1600	G → A	100	M377I	NP
	2357	C → A	100	H630N	NP
	6231	T → C	100	S65P	GP
	6774	T → C	100	S246P	GP
	7368	T → A	100	L149Q	GP
	10342	$(A)_{6\rightarrow 7}$	100		VP30/VP24 intergenic region
	10493	C → T	100	T50I	VP24
	10979	A → T	100	K212M	VP24
	14380	T → C	100	F934L	L
	15301	A → G	9.2	K1241E	L
	16174	A → G	100	I1532V	L
EBOV/Makona-MA-PP3	256	C → T	100		- / NP intergenic region
	683	A → G	100	S72G	NP
	2357	C → A	100	H630N	NP
	4441	C → T	11.6		VP35/VP40 intergenic region
	6231	T → C	100	S65P	GP
	6774	T → C	100	S246P	GP

	10243	G → A	100		VP30/VP24 intergenic region
	10342	$(A)_{6\to 7}$	100		VP30/VP24 intergenic region
	10493	C → T	100	T50I	VP24
	10979	A → T	100	K212M	VP24
	14380	T → C	100	F934L	L
	16174	A → G	100	I1532V	L
EBOV/Makona-MA-PP4	683	A → G	100	S72G	NP
	2357	C → A	100	H630N	NP
	6231	T → C	100	S65P	GP
	6774	T → C	100	S246P	GP
	10342	$(A)_{6\to 7}$	100		VP30/VP24 intergenic region
	10493	C → T	100	T50I	VP24
	10979	A → T	100	K212M	VP24
	14380	T → C	100	F934L	L
	16174	A → G	100	I1532V	L
	18761	A → G	100		L / - intergenic region
EBOV/Makona-MA-PP5	683	A → G	100	S72G	NP
	1967	A → C	15.1	N500H	NP
	2357	C → A	100	H630N	NP
	6231	T → C	100	S65P	GP
	6774	T → C	100	S246P	GP
	9694	T → C	100		VP30/VP24 intergenic region
	10342	$(A)_{6\to 7}$	100		VP30/VP24 intergenic region
	10493	C → T	100	T50I	VP24
	10979	A → T	100	K212M	VP24
	14380	T → C	100	F934L	L
	16174	A → G	100	I1532V	L

Supplemental Table S4. Serial passage of wild-type EBOV/Makona in suckling BALB/c mice. Groups of progressively older suckling BALB/c mice (*n* = 4 to 6) were infected with WT EBOV/Makona by intraperitoneal injection. For each passage, livers were collected, pooled, and used to infect a new set of naïve mice for 11 passages. Corresponding viral loads were measured by RT-qPCR for EBOV L (mean Ct values shown).

	Serial Passage of wild-type EBOV/Makona										
	P1	P2	P3	P4	P5	P6	P7	P8	P9	P10	P11
**Age of Mice (Balb/c)**	3 day (n=4)	3 day (n=6)	3 – 4 day (n=6)	3 – 4 day (n=6)	3 – 4 day (n=6)	3 – 4 day (n=6)	3 – 4 day (n=6)	7 - 8 day (n=6)	14 day (n=6)	14 day (n=6)	14 day (n=6)
**Liver collection**	6 dpi	6 dpi	6 dpi	7 dpi	7 dpi	7 dpi	7 dpi	7 dpi	7 dpi	7 dpi	7 dpi
**Mean Ct value**	23.9	24.3	20.2	20.8	24.3	23.1	22.5	24.5	26.8	24.6	28.0
**% mortality**	0	0	0	0	0	0	0	0	0	0	0
